# Selective ROCK2 inhibition reduces microvascular obstruction but does not reduce myocardial infarction after ischaemia and reperfusion

**DOI:** 10.1016/j.jmccpl.2026.100836

**Published:** 2026-02-06

**Authors:** Lucie Pearce, David He, Derek M. Yellon, Sean M. Davidson

**Affiliations:** The Hatter Cardiovascular Institute, University College London, London, England, United Kingdom

**Keywords:** Cardiac, Ischaemia and reperfusion, Rho kinase, ROCK2, Fasudil, No reflow

## Abstract

**Introduction:**

Myocardial ischaemia/reperfusion (I/R) injury causes infarction, microvascular obstruction (MVO), and haemorrhage. MVO, often driven by vasospasm, lacks effective therapy. The non-selective ROCK inhibitor fasudil, used for cerebral vasospasm, limits infarct size after myocardial I/R, but the roles of individual ROCK isoforms in limiting infarction and MVO remain unclear.

**Aims:**

To determine the contribution of ROCK2 to myocardial and microvascular obstruction and to assess the vasodilatory potential of ROCK2 inhibition.

**Methods:**

ROCK1/2 expression was analysed in rat hearts by RNAscope. Vascular myography assessed arterial responses to ROCK inhibitors. Rats underwent 30 min coronary occlusion and 180 min reperfusion, with ROCK2 inhibitor KD025 (100 mg/kg i.p.) or vehicle administered before reperfusion. Infarct size (%AAR) and MVO (%AAR) were quantified by TTC and Thioflavin S staining, respectively. Infarct size was also compared in WT and ROCK2^+^/^−^ mice.

**Results:**

ROCK2 mRNA was more highly expressed than ROCK1 in both myocardium and coronary vasculature. The dual ROCK1/2 inhibitor, fasudil (10 mg/kg), reduced infarct size (34.5 ± 5.7 vs 55.8 ± 4.7%, P = 0.02, n = 6), whereas the ROCK2-selective KD025 (100 mg/kg) had no effect (43.7 ± 5.5 vs 48.3 ± 4.9%, P = 0.87, n = 8) and also showed no vasodilation ex vivo. ROCK2^+^/^−^ mice were similar to WT. However, KD025 reduced MVO% in rats (21.8 ± 2.5 vs 32.2 ± 1.8%, P = 0.04, n = 8), as did 3 mg/kg fasudil (19.2 ± 4.1 vs 32.2 ± 1.8%, P = 0.01, n = 6).

**Conclusion:**

Dual ROCK1/2 inhibition protects myocardium from I/R injury, whereas selective ROCK2 inhibition or deficiency does not, implicating ROCK1 in infarct limitation. In contrast, ROCK2 inhibition reduced MVO, identifying ROCK2 as a potential microvascular target.

## Highlights

• ROCK2 is more highly expressed than ROCK1 in the heart and coronary vasculature.
• Fasudil, a dual ROCK1/2 inhibitor, reduces infarct size in rats subject to ischaemia/reperfusion.
• The ROCK2-selective KD025 reduces microvascular obstruction, but does not reduce infarct size.
• Infarct size in ROCK2 heterozygous mice is similar to WT.

## Graphical abstract

**Figure:**
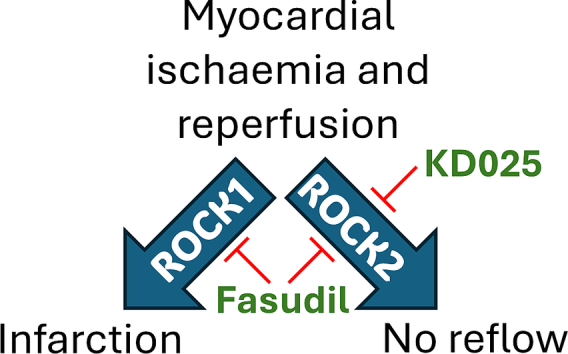

## 1 Background

Although outcomes following ST-elevation Myocardial infarction have vastly improved since the implementation of immediate percutaneous coronary intervention (PCI) [1], microvascular obstruction (MVO) remains a complex problem, with few successful treatment options available [2]. MVO arises due to multiple pathologies following acute myocardial infarction including, capillary damage and rupture, micro-emboli, small vessel vasospasm, immune-platelet complex formation and myocardial oedema and (tissue swelling) [3], [4]. MVO has long been recognised as a lethal form of ischaemia/reperfusion (I/R) injury [5], that leads to ultrastructural changes in the coronary vasculature and microvessels [6]. Traditionally, the field of cardioprotection has concentrated on methods to diminish myocardial I/R injury and myocardial damage, with many successful strategies demonstrated in animal models [7], [8]. However, translation of this approach to humans has been less successful [9]. Therefore, one new approach considered, has been to target the coronary circulation during myocardial infarction [2], [10].

The last two decades have seen increasing interest in inhibitors of Rho-associated protein kinase (ROCK) and their potential for cardioprotection [11], [12]. However, most of these studies have used the non-selective ROCK1 and ROCK2 inhibitor, fasudil [13]. ROCKs are serine/threonine kinases that are highly expressed in the heart, and are associated with a variety of cellular functions including vascular smooth muscle contraction (VSMC) and organisation of the cytoskeleton [14]. There are two isoforms of ROCK, namely ROCK1 and ROCK2. Fasudil inhibits both ROCK1 and ROCK2, in addition to a range of other kinases including protein kinase A (PKA), protein kinase C (PKC) and protein kinase G (PKG). This wide lack of selectivity increases the risk of systemic side-effects including hypotension, following its administration [12], [15]. Fasudil has been shown to reduce infarct size following myocardial I/R in animals in multiple studies [13]. It also improves arterial vasospasm in clinical studies of stable angina and subarachnoid haemorrhage [16], [17]. This vasorelaxant property could potentially contribute to its known mechanism of infarct-size reduction, but could also potentially relieve I/R-associated microvascular obstruction (MVO) [2], [18], [19].

There is growing interest in cardioprotective measures that can mitigate coronary MVO and intra-myocardial haemorrhage (IMH) [20]. IMH, in particular, is associated with worsening survival and heart failure outcomes post myocardial infarction [21], [22]. ROCK inhibitors mitigate vasospasm by reducing vascular smooth muscle cell contraction via modulating the phosphorylation of myosin light chain phosphatase (MLCP) [23], [24]. Fasudil is licenced for the treatment of cerebral vasospasm post haemorrhage in Japan and China and utilises this pathway of vasodilation. Similarly, ripasudil is approved in Japan for the treatment of glaucoma and ocular hypertension [11]. This pathway may also be important during MI, when there is a reduction in nitric oxide, due to endothelial damage [25]. Moreover, there is increasing interest in the role of VSMC reactivity in acute and chronic coronary vasospasm and MVO [26]. This is due to advancing research in coronary physiology which has demonstrated that such coronary reactivity can be detrimental in certain groups of MVO patients [26]. In vitro studies have suggested that ROCK2, but not ROCK1, had a predominant role in VSMC contractility [27], although other authors suggest that there is limited functional difference in the isoforms [11], [14]. Nevertheless, newer and more selective ROCK2 inhibitors such as KD025, have been successfully used in clinical conditions such as graft vs host disease [28], [29]. While KD025 has been investigated in animal models of cerebral I/R injury, it has yet to be examined in studies of myocardial infarction and microvascular obstruction.

Given the established ability of fasudil to limit infarct size, and its potential vasorelaxant properties, we investigated whether it would also improve MVO and IMH (which has not previously been reported in combination in studies of ROCK inhibitors) using an in vivo rat model of I/R. It has recently been proposed that assessment of MVO should be included in the gold standard assessment for future myocardial I/R experiments in vivo [2], [19], [30]. In addition, given the as-yet unexplored potential for ROCK isoform-selective effects in the heart, we investigated the role of selective ROCK2 inhibition in myocardial infarction using KD025, and ROCK2 (+/−) heterozygous mice, to ascertain whether the cardioprotection conferred by ROCK inhibitors is isoform specific.

## 2 Methods

### 2.1 Animals

Adult male Sprague Dawley rats (Charles-River Laboratories) 250–300 g were obtained from the central animal breeding facility at University College London (UCL). Animal handling and experimental protocols were followed in accordance with ASPA 1986 and a license obtained from the UK Home Office. Rats were terminally anaesthetized with 60 mg/kg sodium pentobarbitone via intraperitoneal injection.

A breeding pair of ROCK2 (+/−) mice were obtained from the Centre for Cell Signalling at the John Vane Science Centre, Queen Mary's University of London, and originated from the strain described by Kümper et al. [31], and were maintained at UCL. The background of the mice is C57B6/J OlaHsd. These were compared with wild-type (“WT”) littermate controls.

### 2.2 RNAScope analysis

The RNAScope multiplex fluorescent assay was used to visualise multiple mRNA targets in the same sample by in-situ hybridization. Target mRNAs were identified using isoform-specific probes to ROCK1, ROCK2 and transgelin (TAGLN/SM22α), a marker of differentiated VSMCs. Target probes were designed by ACDBio, with accompanying control probes for cardiac and vascular tissue (ACDBio).

After anaesthesia of rats, thoracotomy was performed to remove the heart under full surgical sedation, (confirmed by absence pedal reflexes) and this was flushed thoroughly with saline via the descending aorta, to remove erythrocytes. Whole heart samples were fixed in 10% neutral buffered formalin for 24 h and transferred to a 70% ethanol solution. Samples were embedded in paraffin and sectioned to 5 μm thickness. Heart sections underwent deparaffinisation with xylene and an ethanol bath series, prior to quenching with 5–8 drops of H_2_0_2_, and target retrieval with RNAScope reagent at 95 °C for 15 min. 100% ethanol was applied to samples, and these were allowed to dry overnight. The RNAScope assay was conducted according to manufacturer's recommendations. Briefly, the target probes were applied to each section (ROCK1, ROCK2, TAGLN), in addition to positive (Polr2a, PPIB and UBC) and negative (dapB) control samples to a concentration of 1:50. Slides were heated for 2 h at 40 °C in a hybridisation oven, before undergoing a three-step amplification process. After the final amplification, samples were washed in wash buffer in preparation for fluorophore application. Opal dyes 520, 570 and 690 (Akoya Biosciences) were reconstituted in 75 μl of DMSO and applied to an individual channel (target probe). Slides were counter-stained with DAPI and mounted with Prolong Gold Antifade Mountant, and cover slipped. Samples were allowed to dry overnight before confocal microscope analysis (Leica microsystems). Images were imported to HALO AI software (Indica labs) as individual channel TIF files. Images were integrated by HALO, which was used to auto-detect and quantify mRNAs of interest within each type of interest. HALO produces an automated H+ score which considers transcript number and intensity for each of ROCK1, ROCK2, TAGLN, as previously described [32].

### 2.3 Vascular myography tissue bath assay

After terminal anaesthesia with pentobarbital, as described above, thoracotomy was performed to remove the ascending aorta. The aorta was placed into ice-cold Krebs-Ringer Solution (NaCl 118 mM; CaCl_2_H_2_0 2.5 mM; d-Glucose 11.1 mM; NaHC0_3_ 25 mM; MgS0_4_ 1.2 mM; KH_2_P0_4_ 1.2 mM; KCl 4.8 mM) before being dissected into 3 mm rings. A tissue bath apparatus (Radnoti) was filled with Krebs-Ringer solution and heated to 37 °C. Aortic rings were carefully mounted onto paired transducer wires above each water bath, then submerged into oxygenated buffer solution and allowed to equilibrate. Transducer wires were connected to Lab Chart 7 Software (AD Instruments) to record change in force in (mN). After 1 h equilibration, aortic rings were pre-constricted with 60 mM KCl to confirm VSMC viability. After KCl washout, 1 μM phenylephrine (PE) was added to constrict, followed by 10 μM acetyl choline (Ach) to confirm endothelial function. After washout to remove Ach, rings were constricted for a third time with 1 μM PE, and when this constriction had plateaued, treated with increasing concentrations of vasodilators (sodium nitroprusside, Ach, fasudil or KD025). Lab-chart curves were analysed, and concentration/relaxation curves were plotted after data normalisation and log transformation using GraphPad Prism 10. A 4-parameter non-linear regression model was fitted to each curve to calculate the LogEC_50_ for the curve.

### 2.4 Rat in vivo myocardial I/R injury

For in vivo experiments, rats underwent general anaesthetic with 100 mg/kg of pentobarbital and were intubated and ventilated throughout. Haemodynamic status was monitored continuously via carotid artery cannulation. Under anaesthesia, surgical thoracotomy was performed, and the left anterior descending coronary artery (LAD) identified within the pericardium. This was ligated for 30 min, and myocardial ischaemia confirmed by the presence of anterior ST-elevation. After 30 min ischaemia, the LAD ligature was released, and the vessel reperfused for 180 min. 15 min prior to reperfusion, either a ROCK inhibitor, or DMSO control, were injected i.p. The dose of 100 mg/kg KD025 was selected based on previous publications that had found this dose to be effective at reducing infarct size in a mouse stroke model [33]. In accordance with previous in-vivo no-reflow models [34], 4% Thioflavin S dye was administered into the systemic circulation at the end of reperfusion, to reveal MVO. Finally, the LAD vessel was re-occluded, and Evans blue dye injected into the systemic circulation, to demarcate the ischaemic area at risk (AAR %) – this provides an objective way to normalize the infarct area to ischaemia area within each heart. The infarct always forms within the AAR, and the MVO region is always within the infarct zone. Myocardium was cut into 2 mm sections and stained with tetrazolium chloride (TTC) to stain live tissue. Infarct size was measured as % AAR. Under UV light, regions not perfused by the Thioflavin S were measured and recorded as regions of MVO%. Regions of IS, MVO and IMH were quantified for each section using Image J software (version 1.54). IMH was defined as the red, haemorrhaged regions, within infarcted regions of myocardium.

### 2.5 Mouse in vivo I/R experiments

In vivo I/R experiments with HET vs WT mice (n = 6 per group) were performed using the protocol described above for rats. However, the duration of ischaemia in these experiments was 40 min, and this was followed by 120 min reperfusion. Myocardial infarct size (%) was assessed as %AAR using TTC as above, with Evans Blue dye to denote the %AAR. Thioflavin S was not used in this protocol.

### 2.6 Proteomic analysis of ROCK isoform abundance

Protein-level abundance of ROCK isoforms in cardiac vascular smooth muscle cells (VSMCs) and cardiomyocytes was assessed using published quantitative proteomics datasets. For cardiac VSMCs, data were obtained from a TMT-based mass spectrometry study in which endothelial cells, pericytes, and vascular smooth muscle cells were isolated from adult mouse hearts and analysed by tandem mass tag (TMT) labelling followed by Orbitrap mass spectrometry (dataset PXD026673) [35] TMM-normalised protein intensities for ROCK1 and ROCK2 were extracted from VSMC samples derived from three independent hearts and used for relative abundance comparisons.

For cardiomyocytes, quantitative proteomic data were taken from a study of acutely isolated adult rat cardiomyocytes analysed by label-free mass spectrometry with intensity-based absolute quantification (iBAQ) [36] Protein-level iBAQ intensities for ROCK1 and ROCK2 were used to estimate relative isoform abundance in cardiomyocytes.

### 2.7 Western blot analyses

Whole hearts from ROCK2 HET and WT mice were lysed in RIPA buffer, and EDTA, protease, and phosphatase cocktail were added to a concentration of 1:100. The tissue was submerged and homogenized using a Potter-Elvehjem grinder, then sonicated for 5 s (Vibracell sonicator) and centrifuged at 10,000 RPM for 10 min. Supernatant was removed and a BCA protein quantification assay performed according to manufacturer's instructions. Prior to electrophoresis, samples were denatured in LDS sample buffer (NuPage) containing β-mercaptoethanol, for 30 min at 80 °C, and used immediately or stored at −80 °C. For ROCK1 and ROCK2 proteins a 4–12% gradient Bis-tris gel was used (Invitrogen). MOPS SDS running buffer (×10) (NuPage) was diluted in ddH_2_0. 20 μg of tissue samples and 7.5 μl of protein ladder (PageRuler Plus, ThermoFisher) were loaded on the gel. Gels were run at 90 V for the first 30 min, followed by 160 V for 75 min. For semi-dry Western transfer, gels were removed and loaded into cassettes with nitrocellulose membrane and submerged in transfer buffer for 90 min at 100 V using a BioRad transfer system. Following transfer, membranes were removed and stained with ponceau red to visualise protein bands. After washing, the membrane was incubated in 10 ml blocking solution (5% Bovine Serum Albumin (BSA)) for 1 h. 1:1000 anti-ROCK1 primary antibody (Abcam AB134181) or 1:1000 anti-ROCK2 primary antibody (Abcam AB125025) was then applied at 1:1000 in 10 ml 5% BSA combined with 1:1000 anti-beta-actin (Abcam AB8226). Membranes were incubated overnight at 4 °C with gentle agitation, then washed and treated with fluorescent secondary antibodies (IRdye 680LT at 1:20000) and (IRdye 800CW at 1:15,000) in 5% BSA and incubated for 1 h. After washing with PBS, fluorescence was visualised using an Odyssey Scanner (LI-COR) and quantified with Image-studio lite v5.5 (LI-COR software).

### 2.8 Statistical analysis

Data is presented as mean ± SEM. Each point represents data from one heart. Data was analysed by Students *t*-test when comparing two groups, or 1-way ANOVA with Tukey post test for more than 2 groups. P < 0.05 was considered significant. Concentration-response data were analysed in GraphPad Prism using the log(agonist) vs. normalised response, Variable slope (four-parameter logistic) model with least-squares nonlinear regression. Data were expressed as percentage relaxation of PE contraction, and each curve was fit independently to obtain Emax and pEC₅₀ values. Pairwise differences in logIC₅₀ values were assessed using an independent-samples *t*-test applied to the difference between parameter estimates, with the standard error of the difference calculated as SE(diff) = √(SE₁^2^ + SE₂^2^). P-values were obtained from the large-sample t (z) approximation.

## 3 Results

### 3.1 Localization of ROCK1/2 mRNA expression in the heart

**Fig. 1:**
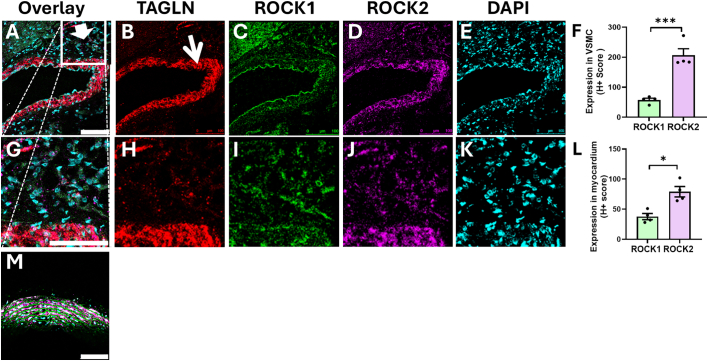
RNAscope analysis demonstrated that ROCK1 mRNA expression was greater than ROCK2 mRNA in rat coronary artery and myocardium. Sections were co-labelled with DAPI (E, K, blue) and an antibody to TAGLN (B, H, red), a marker of vascular smooth muscle cells (VSMC) in the coronary arteries. **(A–F)** ROCK2 mRNA (D, J, purple) is more abundant than ROCK1 mRNA (C, I, green) in coronary artery VSMC (thin arrow) (* P = 0.03, n = 4 rats). **(G–L)** Higher magnification of a region of panels A–E shows that ROCK2 mRNA is expressed more highly than ROCK1 throughout the myocardium (thick arrow) (*** P = 0.0006, n = 4 rats). **(M)** ROCK2 mRNA (purple) is highly expressed in a section of rat aorta (NB: elastin fibre autofluorescence appears green in this image). Scale bars 100 μm (A, C, G) or 50 μm (B, D). Student's unpaired *t-*test. Mean and SEM shown. (For interpretation of the references to color in this figure legend, the reader is referred to the web version of this article.)

In order to confirm and localize the expression of ROCK1 and ROCK2 mRNA in rat hearts, RNAScope imaging was used on cardiac sections from naïve rats. Significantly more ROCK2 than ROCK1 mRNA was detected both in the myocardium and in VSMC of coronary vessels (i.e.: the tunica media) (Fig. 1A–K; H+ expression scores of 58 ± 6 ROCK1 vs 206 ± 22 ROCK2 in myocardium, n = 4, P < 0.001; and 38 ± 5 ROCK1 vs 79 ± 9 ROCK2 in coronary vasculature, n = 4, P < 0.05). The relative expression level of ROCK1 vs ROCK2 mRNA was similar to that observed in a section of rat aorta (Fig. 1M).

Validating these observations at the protein level, quantitative proteomic analysis of adult mouse cardiac VSMCs showed that ROCK2 protein abundance was 3.7 ± 0.1-fold greater than ROCK1 (n = 3 hearts) [35]. In adult rat cardiomyocytes, iBAQ-based quantitative mass spectrometry demonstrated that ROCK2 protein abundance was approximately 11-fold greater than ROCK1 [36]. Comparison with transcript-level data showed close agreement between protein and mRNA ratios in cardiac VSMCs (ROCK2:ROCK1 ∼3.7-fold at the protein level vs ∼3.6-fold at the mRNA level). In contrast, cardiomyocytes exhibited a markedly larger disparity at the protein level (∼11-fold) than at the mRNA level (∼2.1-fold), indicating a substantially enhanced ROCK2 bias at the level of protein abundance. These results demonstrated that ROCK2 is present in the coronary vasculature, and therefore presents a potential target for reducing coronary vascular constriction and MVO during I/R.

### 3.2 Fasudil reduces infarct size after myocardial I/R and attenuates microvascular obstruction

**Fig. 2:**
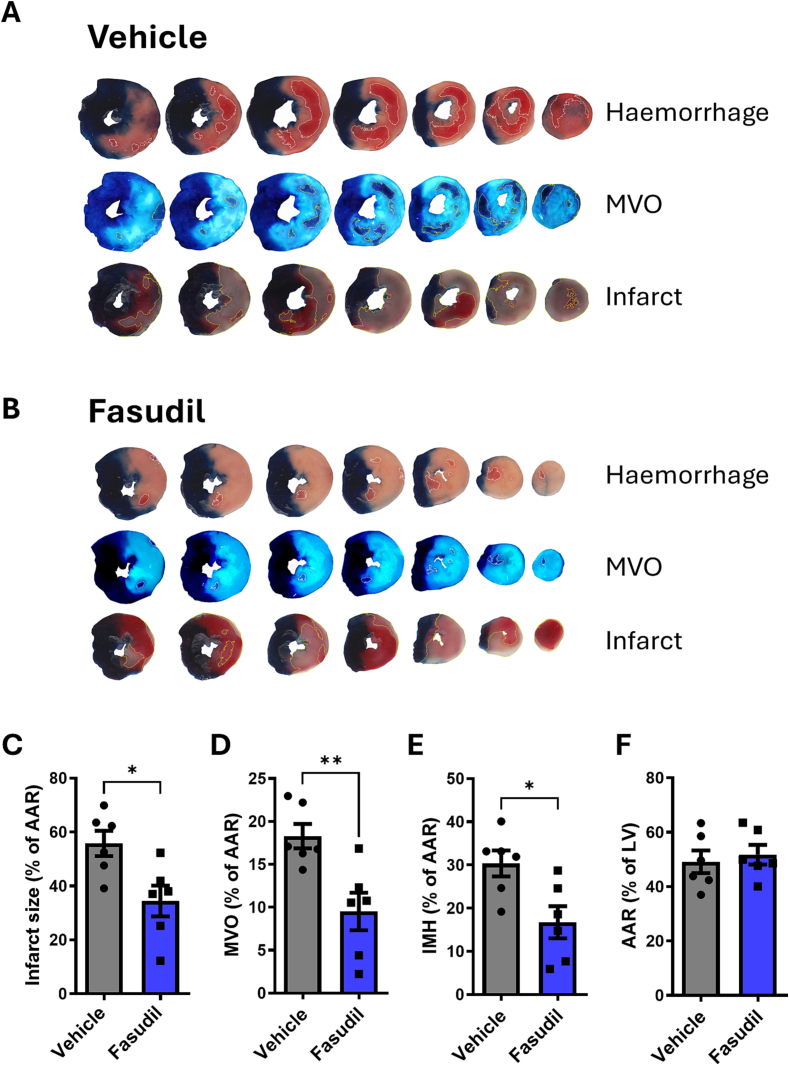
Effects of fasudil on cardiac ischaemia and reperfusion (I/R) injury in rats. **(A,B).** Representative images of heart slices from hearts following I/R, from rats treated with vehicle or fasudil, and stained for haemorrhage microvascular obstruction or infarct. Blue indicates non-ischaemia area (Evan's blue stained). **(C–F)** Fasudil (10 mg/kg) significantly reduced IS% **(C)**, MVO% **(D)** and IMH% **(E)**. There was no significant difference in the area at risk (AAR as % left ventricle) between groups **(F)**. Mean ± SEM is indicated. Students unpaired *t-*test, * P < 0.05; ** P < 0.01, n = 6 hearts per group. (For interpretation of the references to color in this figure legend, the reader is referred to the web version of this article.)

The non-selective ROCK1/2 inhibitor Fasudil was administered to rats subject to I/R, to determine whether it would affect MVO and IMH in addition to infarct size. When administered to rats after ischaemia and prior to the onset of reperfusion, fasudil (10 mg/kg i.p) resulted in significantly smaller myocardial infarct size in comparison to vehicle, respectively (34 ± 5% vs 56 ± 6% n = 6, P < 0.05) MVO (9.5 ± 2.2% vs 18.3 ± 1.4%, n = 6, P < 0.01) and IMH (30 ± 3% vs 17 ± 4%, n = 6, P < 0.05) (Fig. 2A–E). The area-at-risk was comparable between groups (Fig. 2F). However, fasudil was also associated with significant hypotension during reperfusion, reaching a minimum mean arterial blood pressure (MAP) of 52.0 ± 3.9 mmHg (Table 1).

**Table 1:** Effects of fasudil 10 mg/kg on mean arterial blood pressure (MAP, mmHg) during I/R. **P < 0.01.

MAP (mmHg)	DMSO vehicle	Fasudil 10 mg/kg	P value
MEAN ± SEM	88.5 ± 3.4	71.6 ± 3.9	0.008^⁎⁎^
MIN ± SEM	68.8 ± 3.4	52.0 ± 3.9	0.009^⁎⁎^
MAP RANGE ± SEM	47.5 ± 3.4	55 ± 3.9	0.40

### 3.3 Effect of fasudil in an aortic ring vasodilation assay

**Fig. 3:**
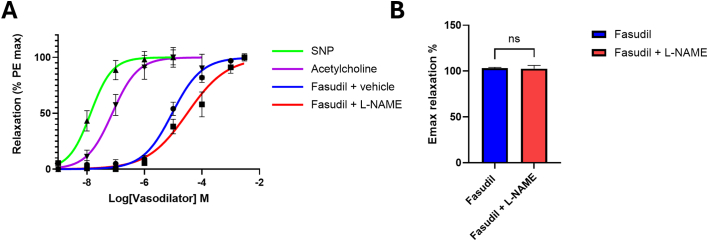
**A.** Fasudil induced relaxation of aortic rings in an organ bath experiment, which was partly dependent on nitric oxide synthase. Fasudil (10^−9^–10^−2.5^ M) induced dose-dependent vasodilation, EC_50_ = 10^–5.0^ M. Fasudil was less potent than sodium nitroprusside (SNP, EC_50_ = 10^–7.9^) & Acetylcholine (EC_50_ = 10^–7.1^). L-NAME significantly decreased potency of fasudil to EC_50_ = 10^–4.5^ M. SNP n = 5, acetylcholine n = 5, fasudil +vehicle n = 8, fasudil +L-NAME n = 8. See methods for description of statistical analysis. All groups were significantly different from each other (see P values in Supplementary table). **(B)** Emax of vascular relaxation by fasudil was not affected by 100 μm L-NAME reduced (ns = non-significant by Students paired *t*-test), n = 8 aortae. Mean ± SEM is indicated. Fasudil (10^−9^–10^−2.5^ M) induced dose-dependent vasodilation, EC_50_ = 10^–5.0^ M. Fasudil was less potent than sodium nitroprusside (SNP, EC_50_ = 10^–7.9^) & Acetylcholine (EC_50_ = 10^–7.1^). L-NAME significantly decreased potency of fasudil to EC_50_ = 10^–4.5^ M. SNP n = 5, acetylcholine n = 5, fasudil +vehicle n = 8, fasudil +L-NAME n = 8. See methods for description of statistical analysis. All groups were significantly different from each other (see P values in Supplementary table). **(B)** Emax of vascular relaxation by fasudil was not affected by 100 μm L-NAME reduced (ns = non-significant by Students paired *t*-test), n = 8 aortae. Mean ± SEM is indicated.

To assess whether fasudil has vasodilatory effects, rat aortic rings in an organ bath were pre-constricted with 1 μM PE and then treated with a range of concentrations of fasudil. As positive controls, an endothelial-dependent vasodilator (Ach) and an endothelial-independent vasodilator (sodium nitroprusside) were used. Fasudil caused vasorelaxation of pre-constricted aortic rings (LogEC_50_ -5.0) although it was less potent than either acetylcholine (Ach) (LogEC_50_ -7.1; P < 0.0001) or sodium nitroprusside (LogEC_50_ -7.9; P = 0.004) (Fig. 3A). L-NAME, an inhibitor of endothelial nitric oxide synthase, significantly reduced relaxation caused by fasudil (LogEC_50_ -4.5; P = 0.05) (Fig. 3A), however it did not significantly affect drug efficacy and maximum relaxation (103.3% vs 102.7%, P > 0.05) (Fig. 3B).

### 3.4 KD025 (a selective ROCK2 inhibitor) reduces no reflow and intramyocardial haemorrhage during myocardial I/R

**Fig. 4:**
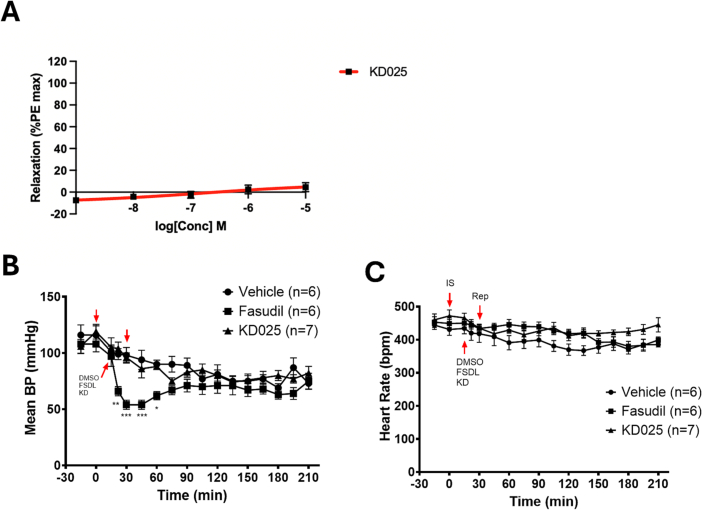
KD025 had no apparent effect on aortic ring relaxation of blood pressure, while Fasudil decreased blood pressure in rats in vivo. **A**: KD025 had no vasodilatory effect on PE-pre-constricted aortic rings ex vivo (n = 5 aortae). **B,C:** Mean arterial blood pressure **(B)** and heart rate **(C)** were measured in anaesthetized rats, following during ischaemia and reperfusion. Rats were administered either vehicle (n = 6 rats), 3 mg/kg fasudil (n = 6 rats) or 100 mg/kg KD025 (KD, n = 7 rats) prior to reperfusion. By 2-way ANOVA and Tukey post test, BP transiently decreased significantly only in the fasudil group (* P < 0.05, ** P < 0.01, *** P < 0.001 vs vehicle).

We considered the possibility that vasodilation by fasudil was mediated by its effects on ROCK1, and that a ROCK2-selective inhibitor may protect the heart without unwanted effects on blood pressure. First, the vasoactive properties of KD025 were investigated ex vivo, using rat aortic ring myography. Encouragingly, KD025 was not found to be vasoactive in this assay, at concentrations as high as 10^−5^ M (Fig. 4A).

To determine whether an inhibitor of ROCK2 would affect BP we administered 100 mg/kg KD025 to rats prior to reperfusion, and measured the same BP as in rats administered vehicle. Rats administered 3 mg/kg fasudil experienced a severe, though transient, drop in BP during the first 60 min of reperfusion, from 108 ± 7 to 54 ± 4 (Fig. 4B). The heart rate remained similar in each group of rats ranging from 449 ± 6 to 399 ± 4 during reperfusion (Fig. 4C).

**Fig. 5:**
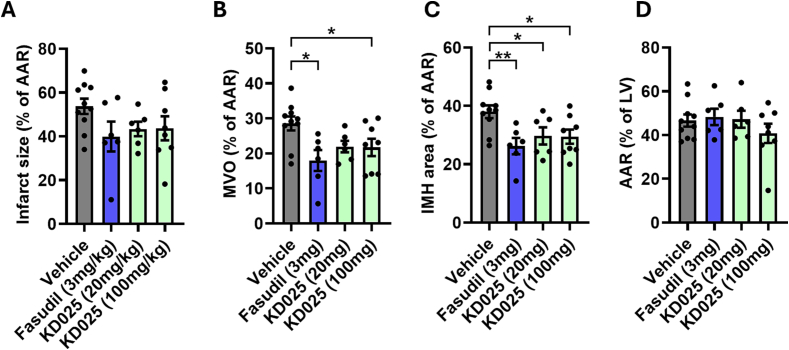
Although KD025 did not reduce infarct size in vivo after ischaemia and reperfusion, fasudil significantly reduced microvascular obstruction (MVO), and both KD025 and Fasudil decreased intramyocardial haemorrhage (IMH). 3 mg/kg Fasudil, 20 mg/kg KD025 or 100 mg/kg KD025 (a selective ROCK2 inhibitor) were administered i.p. 15 min prior to reperfusion in rats subject to 30 min cardiac ischaemia and 2 h reperfusion. At the end of the experiment, infarct size **(A)**, MVO **(B)** or IMH **(C)** were assessed as percentage of the area at risk (AAR). AAR is presented relative to the left ventricular (LV) area (D). Statistics by 1-way ANOVA with Tukey post test. * P < 0.05. ** P < 0.01. Mean ± SEM indicated.

Since 100 mg/kg KD025 did not affect BP, we investigated whether it could limit infarct size following I/R, using the same experimental protocol as above. A control group was also included with fasudil (3 mg/kg), which was a lower dose than used previously, since 10 mg/kg fasudil had been found to induce significant hypotension (Table 1). Both fasudil (3 mg/kg) and KD025 (100 mg/kg) significantly reduced %MVO (from 28.4 ± 2.0% to 20.8 ± 5.8% and 21.7 ± 2.8% respectively) and %IMH (from 38.8 ± 2.1 to 21.8 ± 2.7% and 24.9 ± 3.3% respectively), however they did significantly not reduce infarct size relative to AAR (Fig. 5A–D, Supplementary Fig. 1). 20 mg/kg KD025 had intermediate effects with a significant benefit seen on %IMH (Fig. 5C).

### 3.5 Myocardial infarction size is not reduced in ROCK2 +/− mice

**Fig. 6:**
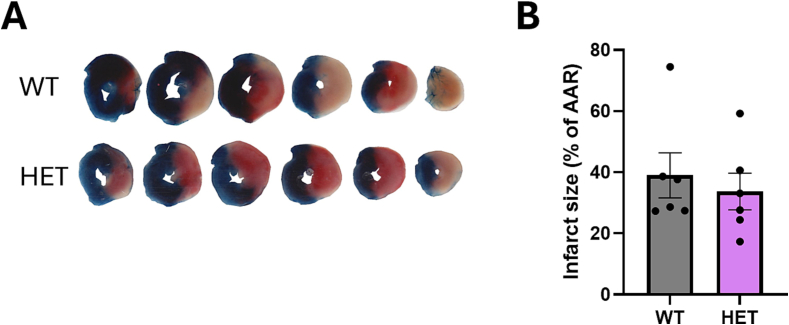
Infarct size (IS expressed as percentage of area at risk or AAR) was not significantly different in WT and HET mice subject to 40 min cardiac ischaemia and 120 min reperfusion in vivo. Representative images of heart slices (A) and infarct size (B). Mean and SEM are indicated. Analysis by Students unpaired *t-*test (n = 6 mice per group).

To investigate whether ROCK2 is an important target in myocardial infarction, heterozygous ROCK2 knockout mice were obtained and bred. Heterozygous ROCK2 mice (“HET”) were used in these experiments because homozygous knockout mice do not survive in utero [31]. Western blot analysis confirmed that HET mice had ∼30% ROCK2 protein remaining compared to WT mice (Supplementary Fig. 2). WT and ROCK2 HET mice underwent I/R with an ischaemic period of 40 min followed by reperfusion for 120 min. No significant differences in IS/AAR% were observed between WT and HET mice (Fig. 6A,B; infarct sizes 34.4 ± 4.5% in WT vs 37.6 ± 6.6% in HET; n = 6; P > 0.05).

## 4 Discussion

In summary, we found that 10 mg/kg fasudil (ROCK1/2i) was able to mitigate infarct size, MVO and IMH following a period of myocardial I/R. This was however, associated with significant hypotension and death of one animal. While the selective ROCK2 inhibitor, KD025, did not reduce infarct size significantly, small reductions in MVO and IMH were observed, although these effects were not as pronounced as with fasudil. Further supporting the hypothesis that ROCK2 is less important than ROCK1 during myocardial I/R, ROCK2 HET mice undergoing myocardial I/R did not demonstrate reduced infarct size compared to wildtype littermates.

Our data demonstrates that the ROCK1/2 inhibitor fasudil is cardioprotective considering outcome measures in vivo, including IS%, MVO% and IMH% at doses of 10 mg/kg. To the best of our knowledge, these are the first preclinical findings to suggest that fasudil may attenuate intra-myocardial haemorrhage following myocardial I/R injury. This is of particular importance considering recent clinical papers that have identified a strong need to limit IMH post MI, given its poor prognosis [21], [37]. Fasudil has been used clinically for several decades in the management of vasospasm associated with intra-cerebral bleeding and in vasospasm associated with stable angina [38], [39]. It is also the focus of current phase III clinical trials for neurological conditions such as Parkinson's disease, and is therefore deemed to be clinically safe as an oral preparation [40]. Nevertheless, fasudil is yet to be investigated in a clinical trial in the context of myocardial infarction and microvascular obstruction. A small retrospective studies has suggested that fasudil is an effective vasodilator in the management of *no reflow* when given during primary PCI [15], but this study did not explore the effects of fasudil on IMH%.

Since ROCK inhibitors can cause vasodilation, the potential risk of hypotension is an important consideration, particularly following I/R when cardiac contractile function may be diminished. Kikuchi et al., reported that some patients became hypotensive following the intra-coronary administration of fasudil [15]. Our results also suggest that fasudil is associated with hypotension when administered i.p. Lowering the dose of fasudil to 3 mg/kg, appears to compromise the infarct lowering effects of the drug (but possibly preserve some microvascular protection). It is likely that fasudil induces hypotension due to its effects as an arterial vasodilator. In addition to inhibition of ROCK (via reducing the active form of myosin light chain kinase) and modifying calcium sensitisation, fasudil is also known to be a calcium channel antagonist [41]. Our results suggest that fasudil is of low potency compared to other well-known vasodilators in the aortic ring myography experiments. However, one advantage of fasudil is that its maximum relaxation effect in aortic rings, does not seem to be inhibited by the eNOS antagonist L-NAME. This may be of particular benefit during acute MI, when vasodilation is required in absence of functional endothelium (and nitric oxide release). In this regard, there is a need for clinically controlled vasodilation in this setting to avoid clinical hypotension.

Although not directly proven here, it might be suggested that fasudil mitigates MVO via an endothelial-independent pathway of vasodilation, i.e. via VSMC-mediated vasodilation. There has been less attention placed on protecting the VSMC during myocardial infarction and coronary injury, in comparison to the numerous agents targeting endothelium. However, VSMC protection was recently identified by Hubert et al. as being integral to minimising coronary circulation injury [26].

The multiple mechanisms by which fasudil attenuates infarction have been reported widely elsewhere [13] and include upregulation of the RISK pathway of cardioprotection and the phosphorylation of AKT [42]. Given that fasudil at doses of 3 mg/kg and 10 mg/kg was able to reduce IMH in vivo, it might be suggested that ROCK1/2 inhibition can alter vascular membrane permeability, possibly via strengthening actin fibres, and so preventing capillary haemorrhage [43]. Further work should be conducted to explore this important mechanism. An important limitation of the above hypotheses is that this work has used aortic rings and not microvasculature, which behave in a different physiological manner, especially during coronary ischaemia [44]. However, basic mechanistic information can be gained, also considering that this work demonstrated that both the coronary circulation and aorta clearly demonstrates ROCK2 mRNA in abundance. This is localised to the VSMC layer, in keeping with previous literature [27].

As previous literature has suggested that non-selective ROCK inhibition, and particularly ROCK1 inhibition, is associated with hypotension (as also demonstrated here), the cardioprotective effects of the selective ROCK2 inhibitor, KD025 were further explored, since KD025 is over two hundred times more selective for ROCK2 than ROCK1 [12], [29], [41]. Importantly, our RNAscope analysis demonstrated that ROCK2 mRNA was present in both the myocardium and coronary circulation.

According to our results, KD025 (selective ROCK2 inhibitor) is less cardioprotective than fasudil in acute myocardial I/R injury. KD025 did not significantly reduce myocardial infarct size, however at 100 mg/kg, there were significant differences observed in MVO/AAR and IMH/AAR. This may suggest that ROCK2 is less important for cardiomyocyte protection but plays a role in protection of the VSMC and its related pathways in MVO and membrane integrity. The fact that KD025 improved coronary circulation outcomes, and not infarct size, might also relate the greater expression of ROCK2 mRNA observed in the vasculature. Wang et al. have suggesting that ROCK2 predominantly regulates VSMC contractility [27]. It was interesting to note KD025's lack of vasoreactivity in PE pre-constricted aortic rings. This may indicate that ROCK1 is more strongly associated with vasodilation in the aorta. However, there is a paucity of selective ROCK1 inhibitors with which to investigate this hypothesis. Lee et al. suggested that KD025 acts directly on migratory machinery of pulmonary endothelial cells to increase membrane integrity and prevent vascular rupture [45]. It is possible that an equivalent process in the coronary microvasculature could be occurring here. A limitation of this experiment is that we did not measure ROCK2 activity, or confirm ROCK2 inhibition by the drug treatments. Analysis of targets downstream of ROCK2 activity is complicated by the fact that ROCK1 and ROCK2 have similar targets.

To further clarify the importance of the ROCK2 isoform in infarct size, ROCK2^+/−^ mice were used in I/R experiments. Homozygous knockout of ROCK2 is lethal, as mice do not survive in utero [31]. Following cardiac I/R, no significant differences in infarct size were observed between wild-type and HET hearts, suggesting that the ROCK2 isoform does not play a major role in cardiomyocyte protection [42]. However, a possible limitation that must be considered is that expression of a single allele of ROCK2 in these mice may be sufficient to retain normal activity. Unfortunately we were unable to assess IMH and MVO in the ROCK2^+/−^ mice as we found these measurements to be unreliable in mice due to the small size of the hearts.

These in-vivo experiments investigated the role of selective ROCK2 inhibitors in cardioprotection, with a particular focus on MVO and IMH, two important clinical parameters, of which at present, there are no gold standard therapies. Here, fasudil demonstrated a novel reduction in IMH, which should be considered in further large-scale clinical trials, given its well-regarded safety profile. We propose this as a potential means of targeting “no reflow”. A limitation of our current experiments is that they were all conducted in male animals, and further work will be required to investigate the effects in females. The newer, more selective, ROCK2 inhibitor KD025, has shown pre-clinical potential in mitigating MVO and IMH, however further studies are required to investigate this mechanism of action. Given the unintended consequences on blood pressure following systemic administration of fasudil, local, intracoronary injection would likely be required in patients.

## CRediT authorship contribution statement

**Lucie Pearce:** Writing – original draft, Investigation. **David He:** Investigation. **Derek M. Yellon:** Writing – review & editing, Supervision, Funding acquisition. **Sean M. Davidson:** Writing – review & editing, Supervision, Project administration, Methodology, Funding acquisition, Formal analysis, Data curation, Conceptualization.

## Declaration of Generative AI and AI-assisted technologies in the writing process

The authors did not use generative AI or AI-assisted technologies in the development of this manuscript.

## Declaration of competing interest

The authors declare no financial interests/personal relationships which may be considered as potential competing interests.

## Data Availability

Raw data is available upon reasonable request.

## Appendix A Supplementary data

Supplementary figures Image 1

Supplementary table Results of statistical analysis of curves in Fig. 3, indicating P values of individual comparisons. Supplementary table
