# Supplementary material for: Selective ROCK2 inhibition reduces microvascular obstruction but does not reduce myocardial infarction after ischaemia and reperfusion
Source: J Mol Cell Cardiol Plus. 2026 Feb 6;15:100836. doi: 10.1016/j.jmccpl.2026.100836 (PMC12907899; doi:10.1016/j.jmccpl.2026.100836)
Supplement: Supplementary file 1 — Supplementary figures [file mmc1.docx]

**Supplementary Figure 1**

Representative images of heart slices with measurement of intramyocardial haemorrhage, microvascular obstruction and infarction in rat heart slices.


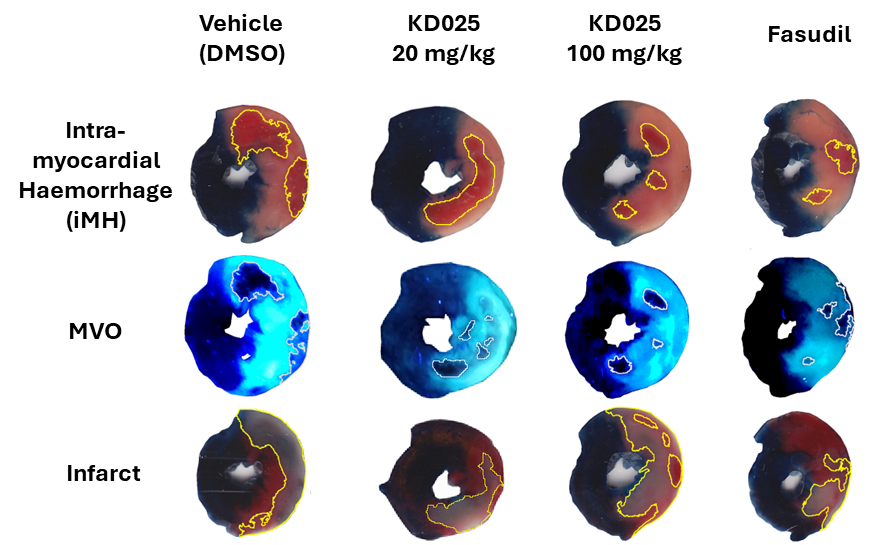


**Supplementary figure 2**

**A)** Original western blot of ROCK1, ROCK2 and beta-actin protein expression in WT and ROCK2 HET mice. Gels were loaded from left to right, with protein extracts from 3 biological replicates of each genotype. The image was obtained by scanning the membrane using a Li-Cor Odyssey fluorescent scanner for 800 nm (IRDye 800CW, GREEN) and 700 nm (IRDye 680LT, RED) channels.

**B,C)** Quantification of ROCK1 **(B)** and ROCK2 **(C)** levels relative to beat-actin.

A


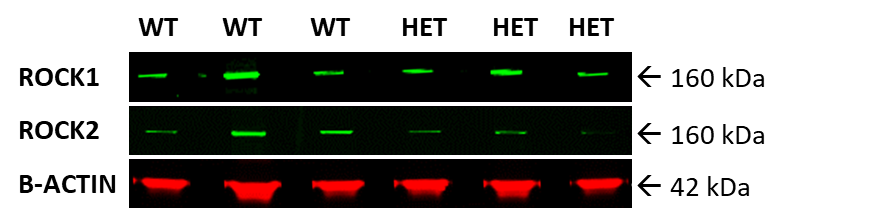


**C**

**B**
