# Supplementary material for: Selective ROCK2 inhibition reduces microvascular obstruction but does not reduce myocardial infarction after ischaemia and reperfusion
Source: J Mol Cell Cardiol Plus. 2026 Feb 6;15:100836. doi: 10.1016/j.jmccpl.2026.100836 (PMC12907899; doi:10.1016/j.jmccpl.2026.100836)
Supplement: Supplementary table — Results of statistical analysis of curves in Fig. 3, indicating P values of individual comparisons. [file mmc2.docx]

**Supplementary table.**

Results of statistical analysis of curves in Figure 3A, indicating p values of individual comparisons.

| Comparison | p value | Significant? |
| --- | --- | --- |
| Control vs L-NAME | ≈0.046 | Yes |
| Control vs SNP | ≪ 0.0001 | Yes |
| Control vs ACh | ≪ 0.0001 | Yes |
| L-NAME vs SNP | ≪ 0.0001 | Yes |
| L-NAME vs ACh | ≪ 0.0001 | Yes |
| SNP vs ACh | ≈0.004 | Yes |
